# Letter to the editor regarding “effects of adjunctive milrinone versus placebo on hemodynamics in patients with septic shock: a randomized controlled trial”: Milrinone in septic shock: hemodynamic improvement without mortality benefit

**DOI:** 10.1080/07853890.2025.2504708

**Published:** 2025-05-20

**Authors:** Muhammad Hasan, Haya S. Ali

**Affiliations:** Dow International Medical College, Karachi, Pakistan

In this issue of *Annals of Medicine*, Tongyoo et al. [1] conducted a randomized controlled trial investigating the effects of adjunctive milrinone on haemodynamics in patients with septic shock. The authors found that milrinone improved cardiac output compared to placebo in patients with persistent tissue hypoperfusion or impaired left ventricular function. However, we would like to emphasize two key findings that have significant implications for the interpretation of these results and their application in clinical practice.

First, despite the improvement in cardiac output, Tongyoo et al. found no significant difference in mortality (28-day, ICU, or hospital) between the milrinone and placebo groups. This finding is consistent with previous research that has questioned the link between hemodynamic improvement and survival in septic shock. For example, the ALBIOS study, a large randomized trial comparing albumin and crystalloid solutions for fluid resuscitation in severe sepsis and septic shock, also found that targeting higher mean arterial pressure and cardiac index did not improve survival [2]. The lack of mortality benefit in the current study, despite the observed improvement in cardiac output, highlights a critical challenge in septic shock management: improving surrogate hemodynamic endpoints does not always translate to improved patient-centred outcomes. This underscores the need for caution when interpreting studies that focus primarily on hemodynamic parameters, and it reinforces the importance of prioritizing outcomes such as mortality in clinical trials.

Second, Tongyoo et al. performed several subgroup analyses to explore whether the effect of milrinone on cardiac output varied across different patient populations. While the authors reported significant cardiac index improvements in certain subgroups (e.g. older patients, males, those with lower baseline cardiac index), subgroup analyses are inherently prone to false-positive findings [3]. As Altman and Bland [3] have explained, subgroup effects are often overemphasized, and statistically significant findings in subgroups should be interpreted with extreme caution, especially when the overall trial shows no effect. The potential for spurious findings is particularly high when multiple subgroups are examined, as was the case in the Tongyoo et al. study. Therefore, the authors’ conclusions regarding the differential effects of milrinone in these subgroups should be viewed as exploratory and hypothesis-generating, rather than definitive. These findings require confirmation in larger, adequately powered studies specifically designed to test these subgroup effects.

In conclusion, while Tongyoo et al. provide valuable data on the hemodynamic effects of milrinone in septic shock, the absence of a mortality benefit and the limitations of the subgroup analyses warrant careful interpretation of the study’s findings. Clinicians should be cautious in extrapolating the observed improvements in cardiac output to improved patient outcomes. Future research should prioritize patient-centred outcomes, including mortality, and avoid overemphasizing subgroup analyses.

## Data Availability

Data sharing is not applicable to this article as no data were created or analysed in this study.
